# Earthquake Trauma, Overgeneral Autobiographical Memory, and Depression Among Adolescent Survivors of the Wenchuan Earthquake

**DOI:** 10.3389/fpsyg.2018.02505

**Published:** 2018-12-10

**Authors:** Qirui Tian, Han Han, Dexiang Zhang, Yuanguang Ma, Jing Zhao, Shouxin Li

**Affiliations:** ^1^Department of Psychology, Shandong Normal University, Jinan, China; ^2^Department of Education, Binzhou University, Binzhou, China; ^3^Department of Primary Education, Jinan Preschool Education College, Jinan, China

**Keywords:** trauma, autobiographical memory, depression, earthquake, adolescent

## Abstract

Trauma has a profound impact on overgeneral autobiographical memory (OGM), which is a risk factor for depression. Violent earthquakes can cause tremendous trauma in survivors. We examined the relationship between earthquake trauma, OGM and depression in adolescent survivors of the Wenchuan earthquake in this study. OGM was assessed using the autobiographical memory test in a sample of adolescent participants who experienced the violent earthquakes in Wenchuan, China, in 2008 and control participants who had never experienced a destructive earthquake. Depression was measured using the Beck Depression Inventory-II in all participants. The results showed that compared with the adolescents with no earthquake trauma, the adolescents with earthquake trauma reported significantly more depression (*d* = 0.49) and overgeneral autobiographical memories (*d* = 0.55). Moreover, when they experienced earthquake trauma, the adolescents with low OGM did not experience more depression, but the adolescents with average and high OGM experienced more depression than the adolescents with no earthquake trauma. This finding indicated that in a non-Western cultural context, adolescents’ propensity toward OGM made them vulnerable to depression after experiencing an earthquake trauma.

## Introduction

In the past decade, several devastating natural disasters, including the Wenchuan Earthquake in China at 2008, the Great East Japan Earthquake at 2011, the Typhoon Haiyan in the Philippines at 2013, and the Nepal earthquake at 2015, had struck the world. Besides human death and injury, such natural disasters also had a severe impact on the mental disorder of survivors. The risk of depression was highly increased after exposure to a natural disaster, and the post-traumatic stress disorder (PTSD) showed high prevalence rate among survivors (Goenjian et al., 2011; Lai et al., 2014).

For survivors, such disasters usually became traumatic events, which could influence the onset of depression. Individuals who experienced a traumatic event, such as burn, traffic accidents, were more likely to be depressed. Substantial body of evidence indicated that exposure to traumatic events could result in disorders (see Williams et al., 2007). There was a high comorbidity between severe post-traumatic reactions and depression after exposure to earthquake trauma among children and adolescents (Pynoos et al., 1993).

On the other hand, individuals who had traumatic experiences exhibited more overgeneral autobiographical memories (OGMs) (see Moore and Zoellner, 2007), which was a kind of autobiographical memory. For example, higher scores on a self-report trauma inventory of the total number of childhood traumas was associated with more OGMs (de Decker et al., 2003). Compared with a control group, adolescents who suffered a burn trauma produced fewer specific memories (Stokes et al., 2004). In addition, adolescents exposed to a war trauma produced significantly fewer specific autobiographical memories than did non-trauma-exposed adolescents (Brennen et al., 2010).

Autobiographical memory refers to one’s memories of personally experienced past events. It contributes to one’s sense of self, one’s ability to approach problems, and one’s ability to orient herself to be in a society (Conway and Pleydell-Pearce, 2000). Overgeneral autobiographical memory characterizes by a tendency to recall categorical events (summaries of repeated occasions, e.g., “I used to go walking in the park every morning”) or extended events (events lasting more than 24 h, e.g., “I borrowed some books on summer vacation”) rather than a specific autobiographical memory (e.g., “I borrowed a book last Sunday morning at 10:00”).

In his trauma hypothesis, Williams (1996) proposed that trauma exposure was critical in the development of overgenerality and suggested that trauma-exposed individuals learned to minimize the negative effect from distressing memories by blocking access to details of such memories or by retrieving these memories in a less specific way. Subsequently, the tendency to truncate the retrieval of trauma-related events could be generalized to much broader domains of autobiographical memories over time, ultimately resulting in a universal OGM retrieval style. Therefore, OGM was viewed as a functional avoidance response to traumatic events and served to regulate intense negative emotions.

OGM of negative events might serve as a marker of depression risk. Adolescents with a current first episode of major depressive disorder retrieved more OGMs than did non-depressed controls (Park et al., 2002), whereas retrieving more specific autobiographical memories seemed to buffer against the impact of negative events on depressive symptoms among adolescents (Hamlat et al., 2015). Additionally, Kuyken et al. (2006) found that adolescents with major depression exhibited a bias toward OGM. Moreover, the risk for depression was associated with a greater tendency to retrieve categorical memories (Hipwell et al., 2011), and OGM predicted depressive symptoms 12 months later (Rawal and Rice, 2012). These studies suggested that OGM retrieval style might be a risk factor for depression in adolescents.

Previous studies in adolescents suggested that trauma exposure was associated with OGM (Brennen et al., 2010) and OGM predicted depression in adolescents (e.g., Sumner et al., 2010; Hipwell et al., 2011; Kuyken and Dalgleish, 2011; Champagne et al., 2016). However, there were conflicting results regarding the relationships among trauma exposure, OGM and depression. Some studies showed no significant correlation between autobiographical memory and depression in adolescents, and only trauma exposure was closely associated with OGM (e.g., de Decker et al., 2003; Brennen et al., 2010). But the mere trauma exposure seemed not to be sufficient to trigger OGM, as in studies of traffic accident victims (Harvey et al., 1998), and cancer patients (Kangas et al., 2005), only people who both experienced trauma and subsequently suffered emotional disturbance, such as depression, had OGM. Other studies had suggested that depressed adolescents with no reported history of trauma exhibited more OGM than both never-depressed adolescents without trauma and depressed adolescents with trauma (Kuyken et al., 2006). Moreover, emotional abuse increased Caucasian adolescents’ depression only when they had higher OGM (Stange et al., 2013). These inconsistent findings might be due to types of traumatic events and emotional disturbance following traumatic events in adolescence. People exposed different traumas often had varying emotional disturbance, such as depression and PTSD (Williams et al., 2007). Such a situation emphasized the need for more studies to investigate the relationships among trauma exposure, OGM, and depression in adolescents. Accordingly, we aimed to examine the relationships between OGM and depression in adolescents with an earthquake trauma in this article.

Furthermore, the cognitive vulnerability-stress model of depression suggested that an individual with cognitive vulnerability was more likely to become depressed than a non-vulnerable individual when she or he confronted a negative event and interpreted the event in a negative manner (Beck, 1987; Abramson et al., 1989). Some studies examined the moderating role of autobiographical memory on the relation between life stress and depression, and found that reduced autobiographical memory specificity moderated the effect of chronic daily hassles on depression (Anderson et al., 2010) and OGM interacted with the occurrence of stressful events to predict depressive symptoms (Gibbs and Rude, 2004). Compared with chronic daily hassles and stressful events, trauma exposure was more severe and harmful, which was very likely to cause depression. An earthquake, especially a violent earthquake, was a severe natural disaster that caused serious harm to individuals who experienced them. Earthquakes resulted in not only physical injuries but also psychological traumas. Individuals exposed to extreme earthquake trauma did not show improvement of their severe PTSD symptoms during three-year interval after their respective traumatic experiences (Goenjian et al., 2000). The effect of earthquake trauma was often lasting and persistent, especially because of the loss of families. The magnitude 8.0 Wenchuan earthquake that struck Sichuan province on May 12, 2008, was the strongest earthquake in 50 years in China and resulted in serious casualties and property losses. Such a severe disaster definitely had a negative effect on people, especially adolescent students, because most of them were at classrooms when the earthquake happened, which resulted in relatively more casualties. The pubertal development at their age stage could exacerbate the negative effect of earthquake. In this study, we examined the relationship between earthquake trauma, OGM and depression by assessing OGM in a sample of adolescents who experienced the Wenchuan earthquake and control participants who had never experienced an earthquake, and investigated whether the relationship between OGM and depression was different between them. Specifically, our hypotheses were as follows: (a) compared to adolescents who had never experienced an earthquake, adolescent survivors of the Wenchuan earthquake would experience more depression; (b) compared to the adolescents who had never experienced an earthquake, adolescent survivors of the Wenchuan earthquake would report more OGMs; and (c) OGM would moderate the relation between earthquake trauma and depression such that higher OGM would made adolescent earthquake survivors experience more depression, but not the adolescents that had never experienced an earthquake. These hypotheses were pertinent to our understanding of the possible etiological mechanisms that might underlie the development of depression in adolescents who experienced earthquake trauma.

## Materials and Methods

### Participants

Ninety-three participants, including 47 participants (26 females, *M*_age_ = 14.77, *SD*_age_ = 0.56) in the earthquake trauma group (ET) and 46 participants (26 females, *M*_age_ = 14.60, *SD*_age_ = 0.39) in the never-experienced earthquake (NEE) group, were recruited in 2010. The two groups matched on age, gender and educational level (both were at the second year of the middle school), and also lived in regions that were similar on economic development. There was no significant difference in age *t*(91) = 1.64, *p* = 0.10, and gender ratio χ^2^(1, *N* = 93) = 0.10, *p* = 0.75 between the two groups.

### Procedures

This study was conducted in accordance with the recommendations of Shandong Normal University ethical guidelines and the Declaration of Helsinki. The protocol was approved by the Human Research Ethics Committee of Shandong Normal University. The written informed consent from all participants was provided by their caregivers.

To identify the earthquake trauma group, 60 students from Beichuan Middle School in Beichuan County, the center and the most seriously damaged area of the Wenchuan Earthquake^1^ in Sichuan Province, China, were evaluated through a self-edited Earthquake-Related Experiences Questionnaire (EREQ) by their head teachers, who had taught these students for more than 1 year and knew the students well. Then students confirmed the information provided by their teachers. The students, who met at least three criteria in the EREQ, were included as the earthquake trauma group. Specifically, 17 participants met the three criteria (36%), 19 met the four criteria (40%), 8 met the five criteria (17%), and 3 met the six criteria (6%). Participants in the NEE group were from Changqing Middle School in Changqing County in Shandong Province, China, where no earthquake had occurred for 20 years. After the participants were chosen, they completed the BDI-II in their own classrooms. Next, the participants individually completed the AMT in a quiet cubicle. Finally, the participants were offered a gift.

### Materials

#### Earthquake-Related Experiences Questionnaire (EREQ)

Earthquake-related experiences questionnaire developed by ourselves was used to access the earthquake-related trauma. It included six items measuring (1) serious casualties, (2) witnessing death, (3) touching a corpse, (4) being buried, (5) physical harm, and (6) family loss with a *yes/no* choice.

#### The Beck Depression Inventory-II (BDI-II)

The BDI-II was a 21-item self-report questionnaire to measure current levels of depression (Beck et al., 1996). The Chinese version of BDI-II was translated and revised. Because of the young age of the participants, we deleted the item of “Loss of Interest in Sex” in this study. All of the participants were asked to indicate how often they felt depressed during the past 30 days. All of the items were scaled from 0 (not present) to 3 (severe). In this study, the total score of the questionnaire ranged from 0 to 60, with higher scores indicating higher levels of depression. The internal consistency was 0.80.

#### Autobiographical Memory Test (AMT)

The AMT (Williams and Broadbent, 1986; Roberts and Carlos, 2006) included 6 positive (exciting, amity, peace, gentilesse, carefree, and comfort), 6 negative (tragedy, distracted, hurt, bad, irksome, and fault), and 6 neutral (grass, return, piano, uncle, onion, and library) cue words to measure OGM. All the cue words were presented to the participants on cards and shown in a repeated sequence of a positive word, a neutral word, and then a negative word. To ensure that the participants understood the task, three practice words (like, brave, and happy) were administered first. When a cue word was shown, the participants were asked to retrieve a personal memory regarding a specific place at a specific time within 60 sec. All responses were videotaped, transcribed, and coded as three types of memory. A specific memory was defined as a recollection of an event that occurred at a particular time on a specific day. An OGM was defined as the recollection of repeated events or memories of events that lasted longer than 1 day. No memory was defined as only semantic associations or future thinking without mention of any specific and/or repeated events. Omission was defined as no response. Another rater coded the videotapes of 15 randomly selected participants to assess the inter-rater reliability, which was found to have a kappa of 0.87. The proportion of OGM in the AMT was calculated by excluding the omissions and then analyzed.

## Results

### Depression and OGM Differences Between Groups

The Means and SDs of the depression and OGM proportions were shown in Table 1. An independent sample *t*-test was performed between the ET and NEE groups on depression. The ET group had significantly higher level of depression compared to the NEE group *t*(91) = 2.38, *p* = 0.02, *d* = 0.58.

**Table 1:** Depression and proportion of overgeneral autobiographical memory (OGM) of the Earthquake-trauma Group (ET) and the Never-Experienced Earthquake Group (NEE).

		ET(*n* = 47)		NEE(*n* = 46)	
		*M*	*SD*	*M*	*SD*
Depression		17.77	7.48	14.50	5.62
OGM proportion	Positive cues	0.16	0.08	0.15	0.09
	Neutral cues	0.17	0.10	0.13	0.09
	Negative cues	0.16	0.08	0.12	0.08
	Total	0.50	0.14	0.40	0.21


To assess whether the two groups differed in terms of OGM, an independent sample *t*-test was performed between the ET and NEE groups on OGM proportion. The ET group had significantly more OGMs than the NEE group in the total proportion *t*(91) = 2.66, *p* = 0.009, *d* = 0.48. The ET group had significantly more OGMs compared to the NEE group for the negative cues, *t*(91) = 2.46, *p* = 0.02, *d* = 0.49 and the neutral cues *t*(91) = 2.17, *p* = 0.03, *d* = 0.46. However, there was no significant difference between the ET and NEE groups for the positive cues, *p = 0.*40.

### The Relationship Between Earthquake Trauma Exposure, OGM and Depression

Using a Pearson’s product-moment correlation coefficient, we found no significant correlation between OGM and depression (*r* = 0.08, *N* = 93, *p* = 0.45); however, a significant correlation was found between the earthquake trauma and depression (*r* = 0.24, *N* = 93, *p* = 0.02), and OGM (*r* = 0.27, *N* = 93, *p* = 0.009).

### OGM Moderates the Relationship Between Earthquake Trauma Exposure and Depression

**FIGURE 1:**
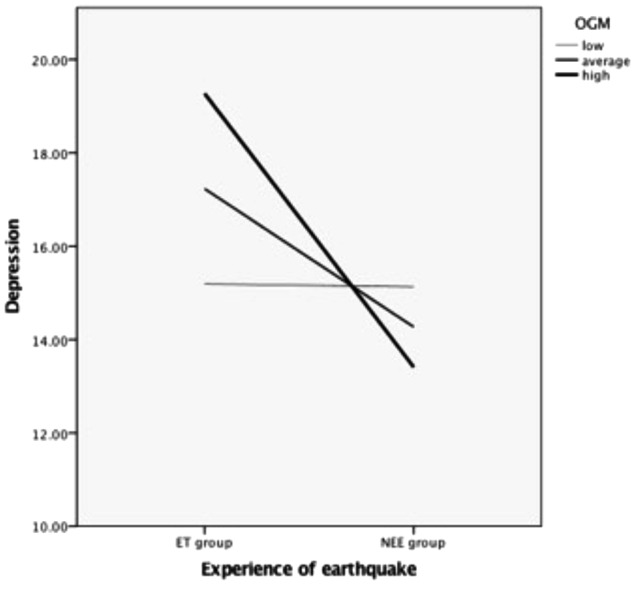
The moderating effect of OGM on depression between the Earthquake Trauma (ET) group and Never Experienced Earthquake (NEE) group.

A SPSS macro (PROCESS) designed by Hayes (2013) was used to examine the moderating effect of OGM on the relationship between earthquake trauma and depression. It revealed that the model as a whole was significant, *F*(3,89) = 3.59, *p* = 0.02, *R*^2^ = 0.10. Specifically, OGM showed no significant effect on depression, *b* = 0.08, *t*(89) = 0.92, *p* = 0.36, 95% confidence interval (CI) = [-0.21,0.58], but the earthquake trauma had a significant effect on depression, *b* = -0.96, *t*(89) = -2.10, *p* = 0.04, 95% CI = [-5.76, -0.16]. Further, the moderating effect of OGM on the relationship between earthquake trauma and depression was significant, *b* = -0.88, *t*(89) = -2.22, *p* = 0.03, 95% CI = [-1.66, -0.09]. A simple slope analysis revealed that for participants with low OGM, there was no relationship between the earthquake trauma and depression, *b* = -0.06, *t*(89) = -0.03, *p* = 0.98, 95% CI = [-4.12, 4.00], but for participants with average OGM, *b* = -2.96, *t*(89) = -2.10, *p* = 0.04, 95% CI = [-5.76, -0.16], and high OGM, *b* = -5.86, *t*(89) = -3.27, *p* < 0.01, 95% CI = [-9.43, -2.29], the earthquake trauma made them experience more depression (see Figure 1).

## Discussion

This study aimed to examine the relationship between earthquake trauma, OGM, and depression in adolescents who experienced a violent earthquake in a Chinese sample. First, it revealed that adolescents exposed to an earthquake trauma experienced more depression than adolescents with no history of experiencing a violent earthquake. This result was consistent with earlier studies that examined the prevalence of depression after exposure to other types of trauma (e.g., physical injury, O’Donnell et al., 2004), and with studies consistently showed that individuals who experienced a trauma were more likely to be depressed (Pynoos et al., 1993; O’Donnell et al., 2004).

Second, it showed that the adolescents exposed to an earthquake trauma recalled more OGMs than adolescents with no history of experiencing a violent earthquake, which was consistent with earlier studies on the relation between OGM and other trauma exposure, including childhood abuse (de Decker et al., 2003), burn (Stokes et al., 2004), and war (Brennen et al., 2010). The participants in this study were between 11 and 13 years of age (during late childhood), when the earthquake occurred in 2008. Therefore, this study’s results were consistent with Williams’ (1996) trauma hypothesis, which stated that children who had experienced trauma relieved their distress by learning to avoid recalling specific trauma-related events. Over time, the tendency to avoid recalling the trauma-related memories generalized to other types of memory, and resulting in an overgeneralized memory retrieval style for autobiographical memories.

Finally, the moderation effect of OGM on the relation between earthquake trauma and depression revealed that the adolescents with average and high OGM, but not those with low OGM, experienced more depression in the earthquake trauma group than those in the never experienced earthquake group. The earthquake trauma might only become evident when accompanied by average and high levels of OGM. One possible explanation was that a tendency toward OGM increased ruminative thinking (Watkins and Teasdale, 2001), which in turn directly influenced depressive symptoms. The tendency of rumination predicted subsequent depression after an earthquake trauma (Nolen-Hoeksema and Morrow, 1991), and OGM partially mediated the relationship between rumination and depressive symptoms (Kong et al., 2015). It was possible that OGM retrieval style might lead participants in our study to engage in rumination after the earthquake trauma, which in turn contributed to their depression. Another possible explanation was that people who had difficulty retrieving specific memories to cues might also have poor problem-solving skills, as impaired problem-solving skills were a function of OGM retrieval in the context of depression (Goddard et al., 1996). When exposed to an earthquake trauma, adolescents with higher OGM might not manage and resolve the problems caused by the traumatic experiences, because of their limited problem-solving skills, further resulting in more depression.

In addition, the cognitive vulnerability-stress model of depression suggested that an individual with cognitive vulnerability was more likely than a non-vulnerable individual to become depressed when confronted with a negative event (Beck, 1987; Abramson et al., 1989). OGM constituted a cognitive vulnerability that predisposed individuals to depression after experiencing negative events. The earthquake in our study was definitely a severe negative event and might make people, especially those with OGM, and show depressive symptoms. As such, adolescent participants in our earthquake trauma group experienced more depression, when they showed higher OGM, than those in the never experienced earthquake group. Such an effect of OGM provided some clinical implications for treating depression and trauma. That is, when treating earthquake survivors, an attempt to regulate their autobiographic memory and to decrease their level of OGM might help them to recover from the disaster.

This study had some limitations that needed to be addressed in future studies. First, when selecting the participants in the earthquake trauma group, we chose them according to the evaluation of the head teachers, who knew the participants well in both their family situation caused by the earthquake and academic performance at school, and but not based on the self-reported evaluation of participants themselves. Although such strategy made participants avoid ruminating the nightmare of earthquake again, it limited the conclusions that one can draw about the onset of earthquake trauma. Second, we did not measure participants’ traumatic experiences before earthquake (such as childhood abuse), which influenced people’s depression (Kuyken and Brewin, 1995) and OGM (Kremers et al., 2006), between ET and NEE groups. As such, our findings should be interpreted with caution, although they were consistent with previous findings (e.g., Pynoos et al., 1993; de Decker et al., 2003; O’Donnell et al., 2004; Stokes et al., 2004; Brennen et al., 2010).

In summary, the current study revealed that adolescents exposed to earthquake trauma reported more depression and more OGM, and provided support for the moderating role of OGM on the effect of traumatic experiences on depression within an earthquake trauma sample in a non-Western cultural context. This finding highlighted that when suffering an earthquake trauma, people’s propensity toward OGM was a vulnerable factor for depression.

## Author Contributions

QT wrote the article. HH and DZ analyzed the data. YM gave suggestions on writing. JZ collected the data. SL designed the study and modified the article.

## Conflict of Interest Statement

The authors declare that the research was conducted in the absence of any commercial or financial relationships that could be construed as a potential conflict of interest.

## Footnotes

1 See https://en.wikipedia.org/wiki/2008_Sichuan_earthquake
